# Diabetes Technology Accessibility in Deaf and Hard of Hearing People With Diabetes

**DOI:** 10.1155/jdr/9944722

**Published:** 2025-10-08

**Authors:** Michelle L. Litchman, Karissa Mirus, Lorne Farovitch, Andrew Bray, Nancy A. Allen, Catherine Elmore

**Affiliations:** ^1^College of Nursing, University of Utah, Salt Lake City, Utah, USA; ^2^Global Deaf Research Institute, Austin, Texas, USA

## Abstract

**Background:**

Deaf and hard of hearing (DHH) populations face higher rates of diabetes and systemic barriers accessing diabetes technology. Effective use of diabetes technology relies on sensory (e.g., visual and audible) input and interpretation by the user. This study evaluates the accessibility of continuous glucose monitoring systems (CGMs) and insulin pumps for DHH individuals.

**Methods:**

A 2-h focus group was conducted to comprehensively evaluate the accessibility of two CGM and six insulin pumps in a sample of DHH individuals during an interactive, hands-on session. Participants used an investigator-developed 6-point Likert-like scale to score audible, haptic, and visual alarm features for each device and provided additional qualitative feedback that was captured. Observational field notes and team debriefing notes were analyzed using a thematic qualitative approach.

**Results:**

Nine diverse DHH participants living with diabetes consistently scored all devices as poor across each alarm feature. Median scores for audible, haptic and visual alarms for CGM were 2, 2.6, and 1.2 and for insulin pumps were 0.5, 2, and 0.7, respectively. The overall qualitative theme was that the evaluated diabetes technology devices were not designed to be accessible for DHH individuals. Participants noted that CGM and insulin pump companies fell short in providing audible, haptic, and visual alarms and provided several important recommendations.

**Conclusion:**

This study highlights the importance of diabetes technology companies including the creative and diverse perspectives of DHH people in diabetes technology design and testing to optimize accessibility. This inclusive approach fosters innovation by integrating universal design principles that can ultimately enhance the experience for all users.

## 1. Introduction

Deaf and hard of hearing (DHH) populations experience diabetes, including conditions that are more likely to be insulin-requiring, such as Wolfram syndrome, maternally inherited diabetes and deafness, Rogers syndrome, and Hermann syndrome, at higher rates than hearing counterparts [1, 2]. Diabetes technology, such as continuous glucose monitoring systems (CGMs), provides real-time glucose readings and, when used with an insulin pump, allows for more precise and flexible insulin dosing. These diabetes technologies help individuals increase the time spent in the recommended glucose range of 70–180 mg/dL while minimizing the risk of hypoglycemia. The American Diabetes Association posits that diabetes devices should be offered to people with diabetes, selecting devices that are individualized to the person's needs and recommending that individuals receive initial and ongoing training [3]. However, DHH populations experience a myriad of systemic barriers to accessing diabetes technology in ways that are linguistically and culturally relevant [4].

Similar to ethnically/racially and other marginalized groups [5–7], it has been suggested that DHH individuals are more likely to have lower adoption rates of diabetes technology [4] even though DHH regularly use and are comfortable with technology [8]. This is likely the result of several factors. First, American Sign Language (ASL) is the primary language for many DHH people in the United States. Contrary to what many people think, ASL is not “visual English.” In fact, the grammar structure and syntax of ASL and English are quite different [9] and not all DHH people are bilingual, knowing both ASL and English. Therefore, written resources in English may be difficult for monolingual DHH people to understand. Additionally, there are challenges with health literacy [10, 11] due to a lack of access to health information in ASL [12, 13]. DHH individuals also experience lower socioeconomic status as a result of limited educational opportunities, communication barriers that limit job prospects and income [14, 15]. Further complicating diabetes technology access is the fact that DHH individuals also experience challenges obtaining qualified ASL interpreters during clinic visits [16–19] despite the Americans with Disabilities Act, which requires an ASL interpreter to be present when requested [20]. This absence of an ASL interpreter can result in poor patient–clinician communication [21], which can lead to feelings of disempowerment, lack of trust and continuity of care, and misinformation leading to health consequences [22].

Second, DHH people have historically been excluded from clinical trials and technology design [23]. This is likely related to a lack of accessible communication through qualified ASL interpreters in clinical [17, 19] and research settings [23]. As a result, some diabetes technology brands have sensory-related contraindications, indicating that devices are only for those with “adequate hearing” [24–27], thus explicitly excluding DHH individuals from using their products. This exclusion has trickle-down effects, including some health insurance companies not authorizing diabetes technology for DHH individuals. For example, in Colorado [28], Oregon [29], Mississippi [30], and New York [31], Medicaid plans require that the “member or caregiver can hear and view CGM alerts and respond appropriately” to qualify for coverage of these devices.

Finally, clinicians may find language discordance, the language mismatch between the clinician and patient, as a barrier to prescribing diabetes technology to DHH individuals. Diabetes technology use in DHH populations is sparse in the literature, focusing only on case studies of DHH individuals successfully using diabetes technology, such as CGM [32], an insulin pump [33, 34], and a hybrid closed loop system [35].

Human factors and usability are evaluated during the research and design stage of medical technology development. If DHH individuals are not included in the design process, diabetes technology might require adjustments or workarounds to be usable. However, these workarounds do not equate to true accessibility. Diabetes technology is often designed to alarm audibly, alerting the person with diabetes to assess their glucose and/or insulin delivery. However, audible alerts are not effective for many DHH people with diabetes. Many DHH individuals prefer haptic (e.g., vibration, pressure, or temperature) and visual (e.g., flashing lights) alarms. While smartphones have built-in accessibility options for such alarms, not all diabetes technologies are connected to smartphone apps. These alarms can require more power, draining the smartphone battery faster.

Diabetes technology accessibility has been formally evaluated in blind/low-vision populations. Insulin pump and glucometer display screens indicated low vision accessibility due to small font size, low contrast, and reflective displays [36]. Perceptions of healthcare provider use of diabetes technology for blind/low vision populations have also been reported, indicating only 55% of clinicians were aware of the voice-activation feature on Dexcom G6 and found CGM initiation challenging [37]. Diabetes technology accessibility has not been evaluated for DHH populations. The objective of this study was to evaluate the accessibility of CGM and insulin pumps in a sample of DHH individuals.

## 2. Materials and Methods

### 2.1. Design

During a 3-day in-person retreat, Deaf Diabetes Can Together community advisory board (CAB) members were invited to participate in a focus group to evaluate the accessibility of insulin pump and CGM alarms. The CAB was established in February 2022 and had an established relationship with the researchers prior to this focus group. The CAB initially convened to develop a diabetes education intervention for DHH populations. However, during these meetings the CAB had raised several other needs that relate to DHH health, including the need to evaluate the accessibility of health technologies that use alarms. Ethics review was obtained at the University of Utah (#00175705).

### 2.2. Research Team and Reflexivity

Researchers include a DHH woman and DHH woman who are native signers, a hearing woman who is a child of a deaf adult with a diabetes clinical background who is English/ASL fluent, two hearing English-speaking women with diabetes or primary care backgrounds, and a hearing English-speaking man currently enrolled in nursing school and taking ASL classes. All hearing team members have clinical experience working with DHH individuals and have received advanced training on conducting research in DHH populations. As described by another mixed DHH-hearing research team [38], this diverse research team acknowledges that DHH individuals are experts in their own lives. As such, DHH perspectives are valued and prioritized throughout the research process. This team has varied experiences in DHH spaces.

### 2.3. Participants and Setting

To be included, participants had to identify as being DHH, be living with diabetes, and be 18 years or older. It was not required to be using diabetes technology to be included. Participants met in a conference room at a local deaf center for a 2-h focus group in May 2024.

### 2.4. Measures

Participants identified as culturally deaf (indicating they are ASL users), hard of hearing, or deaf–blind. The Diabetes Technology Accessibility Scale was developed by the study team. Each device was scored on a 0–5 scale (0 = *not accessible* to 5 = *fully accessible*) to evaluate the accessibility of the device's audible, haptic, and visual alarms.

Audible alarms came directly from the device and were set at the loudest level possible. Though haptic alarms include vibration, pressure, or temperature [39], devices studied only had vibration. Visual alarms included flashing lights directly from the device or connected to a smartphone. Visual alarms were fixed in brightness and could not be adjusted.

### 2.5. Focus Group

The focus group was conducted in ASL and included two certified ASL interpreters who voiced in English for notetaking purposes. The focus group was cofacilitated by two deaf researchers who are native ASL users and one hearing bilingual ASL/English nurse practitioner/researcher who is a child of a deaf adult. Two additional hearing researchers, including a registered nurse and a nursing student observed participant use of, reactions to, and discussion about each device. A hearing diabetes care and education specialist was also present to help answer more specific device questions.

To evaluate the accessibility of CGM and insulin pumps, each participant received a Diabetes Technology Accessibility Scale score card in a grid format, devices were noted rows and audible, haptic, and visual alarms were noted in the columns. CGM devices assessed were the Dexcom G6 and Eversense. Insulin pump devices assessed were Tandem X2, Tandem Mobi, Medtronic 630 g, Medtronic 770 g, OmniPod Dash, and V-Go. The SmartPen device was the Medtronic InPen. While we had originally planned to assess the Medtronic InPen, we did not have a compatible phone that it could link to, and therefore was not assessed.

During the assessment, each participant held the device in their hand to simulate typical use. The exception was Eversense, which was held up to the skin of the upper arm to simulate typical use. The nurse, nurse practitioner, and certified diabetes care and education specialist went around the room doing a 1:1 visit with each participant to evaluate each device for accessibility. As participants evaluated each device, they recorded a written score and provided additional qualitative feedback on their experience with testing the device, including comments about what would make each device more accessible for them. Field notes by three team members were taken throughout the meeting. These field notes included thick descriptions of participants reactions while testing devices (e.g., facial expressions and body language), written snippets of specific comments as interpreted simultaneously from ASL to English, and summarized verbal or signed and interpreted observations by the researchers in the room. A thorough 1-h long debriefing of the focus group occurred during which all the researchers discussed, with the support of an ASL interpreter, initial impressions and patterns observed during the interactive focus group experience.

### 2.6. Analysis

Descriptive statistics for the Diabetes Technology Accessibility Scale are reported. Qualitative data, including field notes and team debriefing notes, were analyzed using a thematic qualitative approach [40]. Two team members independently coded the data using to ensure consistency and minimize bias. We used a deductive and inductive approach: first, categorizing data based off of a priori audible, haptic, and visual alarms assessments and then, analyzing data based off of participant comments that were unanticipated. To enhance rigor, the analysis followed several phases, including the re-reading of field and team notes, coding of written notes, searching for, reviewing, defining, and then naming themes. Triangulation of data sources and coder agreement were employed to strengthen the credibility and dependability of the findings. The final themes represented salient patterns of responses that addressed the main research question.

## 3. Results and Discussion

### 3.1. Survey Results

Participants included four women and five men (*N* = 9) with a mean age of 53. Two participants were Black, two were Hispanic (Cuban and Mexican), and five were non-Hispanic White. Participants identified as deaf, hard of hearing, and deaf–blind. Though most preferred to communicate in ASL, there was one participant who read lips and voiced in English with some ASL. Five individuals used assisted hearing devices (e.g., hearing aids and cochlear implant). See additional demographics in Table 1.

### 3.2. Diabetes Technology Accessibility Scale

Across CGM and insulin pumps, accessibility scores were lowest for those with audible alarms and highest for those with haptic alarms, though no haptic alarms achieved a score of 4 or higher. Of the two CGM devices assessed, the Eversense received a higher accessibility score for the audible alarm while the Dexcom G6 received a higher accessibility score for haptic and visual alarms. Among the insulin pumps, the OmniPod Dash received the highest audible alarm score, the Tandem Mobi and OmniPod Dash received the highest haptic alarm score, and the OmniPod received the highest visual alarm score. See Table 2 for detail scores on specific devices.

### 3.3. Qualitative Results

The overall theme was diabetes technology was not designed to be accessible for DHH individuals. Participants noted that insulin pump and CGM companies fell short in providing audible, haptic, and visual alarm accessibility in several ways. In addition, participants discussed the creative ways that the DHH community have developed “hacks” to make devices more usable for their needs. Finally, participants raised concerns about the lack of accessible instructions for using and troubleshooting problems with devices. The first three subthemes were based on our a priori assessments. The last two subthemes emerged from the data.

#### 3.3.1. Audible Alarms

For those who used assistive hearing devices, the volume of alarms were too faint for most. Given the varied hearing status and underlying challenges (e.g., tinnitus) for those who had some residual hearing, participants noted different audible needs. One participant indicated, “I can't hear the high frequency, I need a base sound” while another participant stated, “I need different sound choices using the app, Crystals, Hillside [iPhone ringtones], these sounds would cover hard of hearing.” One participant also noted that if alarms cannot be silenced (e.g., severe hypoglycemia) by a deaf user in the settings, they may not be able to hear the alarm even though others can. This could be problematic in meetings, church, or other events where the expectation is a quiet environment. Three participants who were current insulin pump and CGM users all noted that when their hearing aids come off at bedtime, they could not hear any alarms and worry about not being notified of serious hypoglycemia and hyperglycemia in the night. For one hard of hearing individual, the OmniPod alarm sounded similar to tinnitus and triggered their tinnitus to be louder.

#### 3.3.2. Haptic Alarms

Overall participants felt that haptic alarms directly on the skin, which was seen with one CGM device, as beneficial. However, overall haptic alarms were not strong enough to be useful. One participant expressed concern about whether or not Eversense would wake them when sleeping, “I am a heavy sleeper. I take sleeping pills. The vibration isn't strong enough.” Specific to Medtronic 770G, participants noted the haptic alarm was “not long enough and too light.” Regarding the Medtronic 630G, a participant noted “It's so weak I can't feel it. I felt nothing.” To enhance accessibility, there was a recommendation to be able to adjust the strength of the haptic alarm, to lower or higher intensity, as one would do with volume on a radio. For DHH individuals, haptic alarms are beneficial only when the user is holding the smartphone or has it close to their body, as in a pocket. However, when the device is on a table or in a bag, participants reported the haptic alarm would not be useful.

#### 3.3.3. Visual Alarms

A visual alarm directly on the device (Eversense) was not found to be useful in real-world settings since these devices may be positioned on the body in a way that is visually obscured (e.g., under clothing or on the back of an arm). One participant said, “The light on the device isn't friendly, I cannot see the light.” All other visual alarms occurred on a smartphone and participants found this to be sometimes challenging based on if the phone was face down or face up. Similar to haptic alarms, participants recommended having the ability to adjust the strength of the visual alarm to lower or higher intensity.

#### 3.3.4. Alarm Hacks

Once diabetes technology devices were being used in real-world settings, with this specific population, participants had to find creative ways to solve for limitations of the “out of the box” device. To make diabetes technology more accessible, current CGM and insulin pump users were engaging in technology “hacks.” These hacks bridge the gap between the company designed devices and DHH usability. Two CGM users reported using a SugarPixel, which is a secondary glucose display and alert device that links with certain CGMs, at home and work. This secondary display allows them, their family, and/or their coworkers have the visual feedback on current glucose levels. One participant described having three SugarPixel devices in the home so that no matter where he was, his family was cognizant of his glucose level and could help support if needed. Some individuals used a smart watch linked to their CGM, which supported both visual and haptic alarms, to help alert them during the day and while sleeping. One participant noted using the haptic alarm on their iPhone that was strapped to their chest in the night to be alerted to any emergent changes in glucose. Participants noted using existing deaf alarms (e.g., flashing lamp alarm) and questioned whether or not diabetes technology could seamlessly integrate. One participant asked, “could these [devices] be connected to a vibration disk under my pillow?” Furthermore, all users reported concern about the cost of these additional devices that are not covered by health insurance.

#### 3.3.5. Technology Instructions and Troubleshooting

A few participants noted interest in certain CGM devices; however, their phones were not compatible. Current CGM and insulin pump users described concern about package instructions and diabetes technology company websites not being available in ASL. There was a recommendation to include QR codes that link to videos that provide descriptions of the written English instructions in ASL. While the V-Go received the lowest scores across all alarms, the functionality to deliver insulin via button click was noted as an insulin pump device that would be accessible to deaf–blind individuals if other insulin pumps could not be tailored to their needs.

## 4. Discussion

This is the first study to evaluate alarm accessibility in diabetes technologies specific to DHH individuals. Alarm accessibility is a critical function for DHH people with diabetes, and yet participant accessibility scores and qualitative feedback demonstrate that none of the CGM and insulin pump devices had adequate alarm accessibility for the DHH users. Below, we describe recommendations for industry and clinicians.

These findings suggest the need for including DHH individuals in the development of diabetes technology devices. Universal design, also known as inclusive design, considers the needs of all people, not just the dominant majority. To create inclusive technology, diverse individuals should be included in the creation, development, and execution of diabetes technology [41]. Diabetes technology development would benefit from “deaf gain,” a term that denotes a DHH individuals' capacity to provide novel insights and perspectives that are less commonly identified by the majority of hearing individuals [42] and supports universal design. Including DHH individuals in the research and development of diabetes technology would avoid the need for “hacks,” which are not considered valid forms of accessibility [4]. As new devices or new iterations of existing devices are developed, we encourage diabetes technology companies to include DHH individuals in their evaluations of how devices and their alarms could be enhanced for the benefit of all users.

In addition to diabetes technology alarms being more accessible, the ability to troubleshoot the device needs to be more accessible to DHH populations. Some diabetes technology companies have started to expand package instructions beyond English to include Spanish [43, 44], French [45], German [46], and other written languages; however, instructions are not yet in ASL. Healthcare provider bias and lack of resources to initiate and sustain diabetes technology for DHH people with diabetes may impact provider confidence in prescribing standard-of-care diabetes technology [7]. This is especially true if device instructions and troubleshooting are unavailable in ASL. We recommend that diabetes technology companies include ASL instructions and troubleshooting instructions developed in collaboration with certified deaf interpreters to ensure accessibility and clarity for ASL users.

Given existing hearing aids with Bluetooth functionality and new indications for some wireless earbuds that provide a clinical-grade hearing aids feature (e.g., Airpods Pro) [47], DHH with residual hearing may benefit from diabetes technology that allows for alarms through a smartphone. However, hearing aids and other hearing assistive devices (e.g., cochlear implants) are removed at bedtime. Diabetes technology is especially helpful during sleep at alerting individuals to dangerous hypo- and hyperglycemia events. DHH individuals have developed creative hacks, such as a haptic alarm by wearing a smartwatch or physically securing a phone to the chest. Throughout history, deaf designers have created alarms to manage daily life (e.g., vibrating disk under the pillow alarm clock and flashing lights in response to a crying baby or smoke alarm) [48]. These additional devices have ways to be linked to existing diabetes technology. Furthermore, these additional devices should be covered by health insurance given that alarms are an essential function and should be functional and accessible to all users [4].

The spectrum of hearing loss varies among DHH individuals [49], as such it is important to tailor a CGM and/or insulin pump prescription based on the individual needs [3]. This requires clinicians, such as diabetes care and education specialists, to have to fully functioning devices, not just demonstration devices that have limited features, for patient testing in clinical settings. This way, all features can be assessed by the DHH person with diabetes to make an informed decision on what device will best work for them. As recommended in the blind/low vision community [37], clinicians should be open to prescribing diabetes technology to DHH individuals and be aware of existing accessibility features, their limitations. Furthermore, clinicians would benefit by being aware hacks DHH individuals are using to ensure alarms are effective in alerting them to changes in glucose or insulin delivery.

A major strength of this study was the inclusion of diverse deaf, hard of hearing, and deaf–blind individuals in the technology assessment. Another strength was having individuals who were current users of CGM and insulin pumps, which allowed us to gain insight on hacks and recommendations to make devices more accessible. Additionally, the diverse deaf-hearing research team included several perspectives when collecting and analyzing data. This approach addresses power dynamics often seen in DHH research [50]. While some may note the potential bias given the previous relationship between the CAB and research team, there is also strength in this long-term trusting relationship. A marker of community-engaged research is ongoing engagement and working to address community needs [51]. The need to evaluate access to diabetes technology was initially identified by the CAB and they served as the initial participants who performed the evaluation. Future research should expand to include a larger sample of DHH individuals, including deaf, hard of hearing, and deaf–blind, who are both naïve and current users of diabetes technology. A limitation of the study is the fact that not all FDA-approved CGM and insulin pumps were evaluated in this study due to a lack of availability. Future studies should examine all FDA-approved devices, including alarm accessibility with various smartphone brands, and secondary devices (e.g., smartwatch and SugarPixel).

## 5. Conclusion

The use of CGM and insulin pumps is state-of-the-art for people living with diabetes. DHH people with diabetes have been systematically left out of the research and design of these devices, resulting in suboptimal levels of accessibility, a lack of insurance coverage, and the use of hacks to improve functionality. To increase accessibility and, thus, improve optimal health, diabetes technology companies need to include the creative and diverse perspectives of DHH people in diabetes technology design and testing. This inclusive approach fosters innovation by integrating universal design principles that can ultimately enhance the experience for all users.

## Figures and Tables

**Table 1:** Demographics.

	**N** = 9**(%)**
Gender	
Man	5 (55.6)
Woman	4 (44.4)
Age (mean, range)	53 (43–65)
Race/ethnicity	
Non-Hispanic, White	5 (55.6)
Hispanic/Latino	2 (22.2)
Black	2 (22.2)
Deaf identity	
Culturally deaf	3 (33.3)
Hard of hearing	4 (44.4)
Deaf–blind	2 (22.2)
Preferred communication	
American Sign Language	8 (88.9)
Reading lips/voicing	1 (11.1)
Assisted hearing devices	5 (55.6)
Diabetes type	
Type 1	5 (55.6)
Type 2	4 (44.4)
Current diabetes technology	
CGM user	7 (77.8)
Insulin pump user	5 (55.6)
Smartpen user	0 (0)
None	2 (22.2)

**Table 2:** Diabetes technology alarm accessibility.

	**Audible alarm** **mean (mode, min–max)**	**Haptic alarm** **Mean (mode, min–max)**	**Visual alarm** **Mean (mode, min–max)**
CGM			
Dexcom G6	1.5 (0, 0–5)	3.8 (0, 0–5)^a^	2.3 (0, 0–5)^b^
Eversense	2.4 (0, 0–5)	1.3 (0, 0–3)^c^	0.1 (0, 0–1)^d^
Average/median	2	2.6	1.2
Insulin pumps			
Medtronic 630G	0.3 (0, 0–2)	1 (0, 0–3)	0.9 (0, 0–5)
Medtronic 770G	0.6 (0, 0–3)	2 (1, 0–5)	0.4 (0, 0–2)
OmniPod Dash	1.4 (0, 0–4)	3.3 (3, 2–5)	1.6 (0, 0–5)
Tandem X2	0.6 (0, 0–5)	2.4 (5, 0–5)	1.4 (0, 0–5)^b^
Tandem Mobi	0 (0, 0–0)	3.4 (3, 1–5)	0 (0, 0–0)
V-Go	0 (0, 0–0)	0 (0, 0–0)	0 (0, 0–0)
Total average	0.5	2.2	0.7
Total median	0.5	2	0.7

^a^Haptic alarm via smartphone.

^b^Visual alarm via smartphone.

^c^Haptic alarm on the device against the skin.

^d^Visual alarm on device.

## Data Availability

The data that support the findings of this study are available upon request from the corresponding author. The data are not publicly available due to privacy or ethical restrictions.
